# Learning to perceive in the sensorimotor approach: Piaget’s theory of equilibration interpreted dynamically

**DOI:** 10.3389/fnhum.2014.00551

**Published:** 2014-07-30

**Authors:** Ezequiel Alejandro Di Paolo, Xabier E. Barandiaran, Michael Beaton, Thomas Buhrmann

**Affiliations:** ^1^Ikerbasque, Basque Foundation for ScienceBizkaia, Spain; ^2^IAS-Research Center for Life, Mind, and Society, Department of Logic and Philosophy of Science, University of the Basque CountrySan Sebastián, Spain; ^3^Centre for Computational Neuroscience and Robotics, Department of Informatics, University of SussexBrighton, UK; ^4^Department of Philosophy, University School of Social Work, UPV/EHU University of the Basque CountrySan Sebastián, Spain

**Keywords:** sensorimotor contingencies, Piaget’s theory of equilibration, dynamical systems, open-ended learning

## Abstract

Learning to perceive is faced with a classical paradox: if understanding is required for perception, how can we learn to perceive something new, something we do not yet understand? According to the sensorimotor approach, perception involves mastery of regular sensorimotor co-variations that depend on the agent and the environment, also known as the “laws” of sensorimotor contingencies (SMCs). In this sense, perception involves enacting relevant sensorimotor skills in each situation. It is important for this proposal that such skills can be learned and refined with experience and yet up to this date, the sensorimotor approach has had no explicit theory of perceptual learning. The situation is made more complex if we acknowledge the open-ended nature of human learning. In this paper we propose Piaget’s theory of equilibration as a potential candidate to fulfill this role. This theory highlights the importance of intrinsic sensorimotor norms, in terms of the closure of sensorimotor schemes. It also explains how the equilibration of a sensorimotor organization faced with novelty or breakdowns proceeds by re-shaping pre-existing structures in coupling with dynamical regularities of the world. This way learning to perceive is guided by the equilibration of emerging forms of skillful coping with the world. We demonstrate the compatibility between Piaget’s theory and the sensorimotor approach by providing a dynamical formalization of equilibration to give an explicit micro-genetic account of sensorimotor learning and, by extension, of how we learn to perceive. This allows us to draw important lessons in the form of general principles for open-ended sensorimotor learning, including the need for an intrinsic normative evaluation by the agent itself. We also explore implications of our micro-genetic account at the personal level.

## Introduction

The sensorimotor approach to perceptual experience (O’Regan and Noë, 2001; Noë, 2004) proposes that at the root of perception there always lies a skilful engagement with the world, with an emphasis on the active connotation of the verb *to engage*. More specifically, perception involves the mastery of the laws of sensorimotor contingencies (SMCs), those regular sensorimotor co-variations that depend on the environment, the agent’s body, the agent’s internal (neural) dynamics, and the task context and norms.

While the proposal has seen no small amount of controversy and debate over the last decade, only recently have attempts been made to produce a formal theoretical framework to express the various claims of the theory, the relation between its proposed concepts, and the implications for experimental and modeling/robotic work. The very notion of SMCs, the core concept of the theory, had no formal expression in dynamical systems terms until a recent mathematical formalization was introduced by Buhrmann et al. (2013).

The goal of the current paper is to proceed along similar lines of theoretical development and examine another central, and heretofore neglected, aspect of the sensorimotor approach: perceptual learning.

What we can perceive seems to rely on the level of embodied know-how that we possess and are able to enact. And yet, although the primary literature discusses various examples of skill acquisition and adaptation to sensorimotor disruptions, it has to date offered no explicit theory of perceptual learning. Such a theory would have to account for how it is possible to perceive anything new if perception relies on pre-existing sensorimotor skills. Moreover, it would also have to account for the seemingly *open-ended* character of human perceptual learning, which is able to exceed any prescribed set of relevant species-ecological criteria by constantly opening up novel domains of significance (e.g., wine-tasting, refined construction of musical instruments). Various developments of the theory have modeled aspects of this process (e.g., Philipona et al., 2003, 2004; Maye and Engel, 2011, 2013). However, it can still be said that the SMC approach lacks theoretical resources to operationally address questions such as:

- By what mechanisms is mastery of SMCs acquired?
- What counts as having acquired sufficient know-how of SMCs? What counts as mastery?
- How is it possible to learn to perceive anything new if perception itself always relies on existing knowledge of SMCs, as the theory claims?
- How do various sensorimotor organizations relate to each other in the same agent?
- What kind of cognitive organizational principles can help sustain SMCs in a flexible, open-ended manner?

In order to address questions like these, SMC theory requires an explicit theory of learning. For reasons discussed below, we propose Piaget’s theory of equilibration as a suitable starting point. In order to demonstrate the compatibility and various complementarities between this and SMC theories, we re-formulate Piaget’s proposal in dynamical systems terms that render it compatible with the dynamical definitions of SMCs (Buhrmann et al., 2013), thus further contributing to the formalization of the SMC approach.

In particular, from the dynamical account of Piagetian sensorimotor learning we derive some principles for the organization and open-ended acquisition of SMCs. There is a vast literature on skill acquisition, cognitive development and learning in psychology (e.g., Speelman and Kirsner, 2005), on plasticity of different kinds in neuroscience (e.g., Sirois et al., 2008), and on models of learning in AI and robotics (e.g., Floreano et al., 2008). However, the objective here is not to provide a thorough review but to focus on feasible ways to solve the problem of open-ended learning in SMC theory at least in the form of general necessary principles and other requirements.

The rest of this paper is organized as follows. The two following sections set the conceptual background, before we turn to a formal development of the theory. We first discuss the central role of understanding within SMC theory. After that we clarify that perceptual learning is centrally important to SMC theory, but that a worked out account of this process is missing; it also highlights the fundamental difficulty of accounting for perceptual learning, in any theory that tightly links perception and understanding, as SMC theory does. Next, we introduce the Piagetian theory of equilibration as a promising candidate from which the missing sensorimotor theory of learning can be developed. Section 5 translates Piaget’s theory into a modern dynamical systems formulation and establishes the strict compatibility with the dynamical operationalization of SMCs provided by Buhrmann et al. (2013). We then derive a list of necessary (but probably not sufficient) principles for open-ended learning architectures. Finally, we discuss what has been achieved, and return to the problems for sensorimotor theory raised at the beginning of the paper.

## The role of understanding in sensorimotor theory

SMC theory emphasizes the claim that all perceiving involves understanding (Noë, 1999, 2004; O’Regan and Noë, 2001). This is not to say that abstract, disengaged reasoning is required. The claim is that the practical, engaged, sensorimotor skills of the whole subject are required, for perception. This is the practical sense of the word understanding, which we will adopt throughout this paper.

To see what this means, consider Kohler’s (1951 [1964]) work, involving long-term adaptation to vision-inverting prisms and lenses. Initially, on putting on such devices, the world stopped making sense to Kohler’s subjects: objects moved around in completely unexpected ways, solid objects no longer even appeared solid, but rather rubbery and distorted, and changeable in size and shape (Kohler, 1951 [1964], pp. 64–65). Nevertheless, for subjects who actively engaged with the world, after a long period of using such devices, the world slowly righted itself. Firstly actions became more and more correct, and eventually perception itself became more and more correct. It is notable that: (a) the adaptations, both behavioral and perceptual, were partial; and (b) they were very situation-dependent: subjects came to perceive correctly only in those situations where they had practice. Note also that Kohler’s subjects often used explicit strategies to react correctly in the initial stages of rehabituation, but that these strategies eventually became implicit, and automatic; and concomitantly, the visual world itself came to look more and more normal.

These experiments indicate that perceptual experience involves practical understanding. In this case, practical, engaged knowledge of how to move and navigate in 3D space is required for experience of 3D space. Note, in particular, that the understanding involved in perceiving space is not just an isolated, automatic happening. Rather, our perception of 3D space (and, on this view, all of our perception) depends on our overall ability to understand and make sense of the world around us. This is why we describe the successfully adapted subject as following “implicit” strategies, to emphasize this continued involvement of whole-agent sense-making, which can become explicit again if required (as Kohler’s experiments show) even in what is normally thought of as “low-level” experience.

## The paradox of perceptual learning

This emphasis on understanding in making perception possible leads to an apparent paradox of learning for SMC theory (and for any theory which tightly links perception to understanding). If understanding is required for perception, how can a subject learn to perceive something new, which they do not yet understand? The contrary claim (e.g., Roskies, 2008) is that the very existence of perceptual learning shows that some experience must outstrip our current understanding.

This is an old philosophical problem. It closely resembles the foundational problem of epistemology as expressed by Plato in the *Meno*. In trying to determine the essence of virtue Socrates admits not knowing what it is but invites Meno to inquire, together with him, into its nature. Meno asks how will they manage to search for something of whose nature they know nothing at all; how will they even recognize it if they find it? Marjorie Grene, following Merleau-Ponty’s and Michael Polanyi’s conceptions of practical, embodied knowledge, comments that the structure of Meno’s problem is particularly puzzling, not to say unsolvable, if we assume that knowledge must be fully explicit (Grene, 1966, pp. 23–24) (Closely related points, to do with the nature of rule-following, have also been made by Carroll, 1895 and Wittgenstein, 1953/2001 §§185–242). However, Grene argues, the problem does not involve any logical contradictions once we admit the possibility of knowledge or understanding having *degrees* of explicitation, from what we can verbalize, to the practical knowledge that we tacitly embody in our everyday skills. The solution to the problem of perceptual learning may be sought in between fully achieved understanding and total ignorance, along a “continuous” space of different degrees of skilful coping with the world.

To resolve this paradox, we need a worked out theory of perceptual learning that builds on the above suggestions. We will now argue that although SMC theory relies on the possibility of perceptual learning, it currently lacks such a worked out theory.

Learning is explicitly mentioned at a few key points in the primary SMC literature. Firstly, as discussed above, the case of adaptation to inverting prisms (Kohler, 1951 [1964]) is seen as an important verification of the theory, especially given that adaptations only occur in the context of active, personal effort by the subject to remaster their visual world. Learning is also important in the discussion of cortical deference vs. cortical dominance given by Hurley and Noë (2003). Here, the issue is that of whether given modalities of experience are always supported by given brain regions. Hurley and Noë discuss several relevant empirical findings, including the very striking experiments on ferret pups carried out by Roe et al. (1990), in which it is shown that the auditory cortex can come to subserve visual experience, given early enough surgical intervention.

Another example of cortical deference occurs in tactile-visual sensory substitution (TVSS). Here, the image from a camera is fed to a 2D array of vibrating touch actuators on the body. In early experiments (Bach-y-Rita, 1967) this was a relatively large array, placed on the subject’s back. In more recent experiments (Sampaio et al., 2001), smaller arrays are situated on the subject’s tongue. Strikingly, subjects who are passively “shown” the world via this system do not learn to perceive anything new. In contrast, those who are allowed to use the camera to actively explore the world can begin to get a sense that the system is providing visual-style access to the world within only a small number of days of training (Guarniero, 1974). Hurley and Noë (2003) argue that there is a distinctively visual phenomenology to this new way of experiencing of the world—even though it is of course not nearly as detailed as normal vision, lacks color, and so on. If this is correct, then this is another case of cortical deference: the areas of the brain that normally subserve touch are now subserving visual experience.

Hurley and Noë’s (2003) general point is that, if SMC theory is correct, cortical deference should be the norm; the brain region is not what matters, what matters is the sensorimotor coordinations which the brain region helps to enable. As long as the subject can learn the relevant, new coordinations, the relevant, new type of experience will emerge.

These discussions show the importance of learning for SMC theory, but they also show that SMC theory does not, yet, explain how perceptual learning can take place, it just presupposes that it can. Thus, we now turn to Piaget’s work, which we believe can be integrated with SMC theory to provide the resolution to this problem.

## Piaget’s theory of equilibration

Within his broader framework of equilibration theory, Piaget has addressed the main difficulties of the problem of perceptual learning (Piaget, 1936, 1947, 1969, 1975; Chapman, 1992, see also Boom, 2009; the relevant literature is large and we only evaluate the theory in its basic form). In this section we briefly go over the central points of Piaget’s research program, which needs to be seen precisely as the quest to determine how abstract and explicit (e.g., conceptual, mathematical, formal, rational) human capacities of understanding stem from early and less explicit forms of sensorimotor organization. The set of developmental transitions that span this continuum are conceptualized under the general notion of adaptation, which itself is seen as a process of equilibration between assimilation and accommodation processes. These notions present us with a suitable candidate solution for the problem of perceptual learning for, as we will see, they assume the possibility of different degrees of explicitation and kinds of understanding.

Following the standard interpretation, by *assimilation* we refer to a process by which an environmental aspect (a perturbation, a new object, or a novel situation, etc.) is integrated, coupled or absorbed into an existing physiological (metabolic, muscular, etc.) or cognitive/behavioral (sensorimotor, perceptual, reflexive) scheme or structure. In Piaget’s famous example (Piaget, 1936, 1947) a baby assimilates the mother’s nipple into a suckling reflex (itself a sensorimotor structure involving a complex of muscular coordinations, proprioceptive, tactile, temperature and taste sensory feedback, etc.). But a propensity to suck does not typically equate to immediate sucking skill. The baby has to learn to “latch on” successfully, to become comfortable with the shape and feel of her own mother’s breast. That is, the baby has to learn new patterns of sensorimotor organization. In Piaget’s notation, an agent’s coordination structure A assimilates an environmental aspect A′, that in turn leads to the coordination B that demands and assimilates B′ in a sequence scheme, or *organization*, expressed as A × A′ → B; B × B′ → C; C × C′ → …. In terms of the example, A can denote suckling and A′ the mother’s breast, B swallowing and B′ the milk, C breathing and C′ air, etc. Note that for Piaget the environment is not a set of pre-existing stimulus conditions that impact on the organism to produce a perceptual or cognitive effect. Only what can be assimilated in an already existing scheme or sensorimotor coordination pattern (i.e., an action or operation of the subject) can be perceived.

By *accommodation* Piaget refers to the process by which the physiological or cognitive scheme or structure is modulated or transformed to facilitate or encompass a not-yet-assimilated aspect of the environment. So, for instance, the suckling sensorimotor coordination of the baby gets progressively attuned to the size, texture and shape of the nipple. So, variations of A get progressively attuned to variations in A′.

*Equilibration* is the process by which a given cognitive or biological organization, as a result of a maturational process or in the presence of an ever changing environment or any internal sources of tension, reaches a new form of organizational stability. Piaget denotes the stability of the organization as the closure of a cycle of sensorimotor engagements: A × A′ → B; B × B′ → C; C × C′ → … → Z; Z × Z′ → A. We refer to this cycle as a sensorimotor scheme or organization. Even though the simple cycle is the paradigmatic case, Piaget allows for the possibility of additional complexity (short-circuits, intersections between cycles, etc., Piaget, 1975, p. 10). He hints at the fact that ultimately a cycle should be understood as conservation of the conditions of viability of an organism or a cognitive system as a whole (ibid., p. 11). In a similar manner to that discussed in current work in the enactive approach (Di Paolo, 2005, 2009; Thompson, 2007; Barandiaran, 2008; Di Paolo et al., 2010; Di Paolo and Thompson, 2014), it is the conservation of a self-sustaining circular organization that can be proposed as grounding aspects of normativity. In this case, the evaluation of whether equilibration occurs (or not) corresponds to the cycle closing back on itself (or not).

A sensorimotor organization that has undergone processes of equilibration will potentially be affected by higher-order internal tensions this may have generated, as well as by new possibilities for action that have been brought about by the adaptive changes involved in its accommodation. For instance, if a baby has acquired a suckling skill and is now presented with a milk-bottle for the first time, she now faces the challenge, and opportunity, of accommodating a new object with new properties (texture, friction, shape, etc.). These differences induce instabilities in the suckling organization scheme. As a result, a new accommodation process is triggered, and repeated exposure to these conditions can lead, by the interplay between assimilation and accommodation, to a new equilibration of the suckling sensorimotor organization and eventually splitting it into two broad categories: A_1_ × A_1_′ → B_1_; B_1_ × B_1_′ → …, corresponding to breastfeeding and A_2_ × A_2_′ → B_2_; B_2_ × B_2_′ → …, corresponding to suckling from the milk-bottle. This new equilibration where milk-bottle feeding is stabilized might open new possibilities for action: the milk bottle affords grasping, new feeding positions are available, etc.

A more radical equilibration process might result from the development of the baby’s muscular and sensorimotor capacities. When the baby starts to grasp objects and bring them to her mouth, the suckling coordination might sometimes be triggered in an assimilation attempt. But subsequent sensorimotor coordinations may be severely challenged if the object, say a puppet, is incompatible with the enacted coordination. The inability to assimilate this new object, and to force an impossible accommodation upon the suckling scheme, may then lead to a higher order equilibration where sensorimotor organizations are now split into suckable and non-suckable (yet graspable, movable, chewable, etc.) patterns. A further equilibration process might result from an increasingly stabilized habit of bringing the thumb or a dummy to the mouth so as to assimilate it into the suckling scheme as breastfeeding becomes less frequent. In turn, this new assimilation A_3_ × A_3_′ might now lead to a modification of the swallowing coordination transforming the original sequence A × A′ → B; B × B′ → … into A_3_ × A′_3_ → B_3_; B_3_ × B′_3_ → … that might later be further transformed into a repetitive biting pattern when teeth start to grow, resulting in A_4_ × A′_4_ → B_4_; B_4_ × B′_4_ → …. These processes of differentiation of sensorimotor organization schemes, their grouping and branching, sequential ordering, etc. lead to higher order equilibrations that are richer in diversity and combinatorial potential than what the previously existing cognitive organization made possible.

Piaget describes two kinds of perturbations that may be encountered by an established sensorimotor or cognitive scheme: *obstacles* (contradictions or disturbances) and *lacunae* (gaps in the current organization) (Piaget, 1975; Boom, 2009). Both types are manifested in the concrete encounter between agent and world regardless of whether they originate from changes in the world or from internal contradictions (for instance, in the case of conflicting sensorimotor schemes). This is important because Piaget’s theory implies, but perhaps does not emphasize enough, that encounters with the world (i.e., obstacles and lacunae) drive equilibration and there is always a possibility that the world may “guide” part of the equilibration process (c.f. Beaton, 2014). The theory might otherwise be interpreted as too internalistic, relying only on the reorganization and adjustment of existing sensorimotor coordinations. Maximal equilibration corresponds to the situation in which a sensorimotor or cognitive organization already anticipates all potential obstacles and lacunae. The latter have no disturbing effect because the cognitive structure has already fully adapted to them. In other words, maximal equilibration would be that (unattainable) state where the enactment of sensorimotor schemes required no further accommodation to the world.

It is important to note that equilibration processes are not limited to agent-environment dynamics. Piaget distinguishes three broad categories of equilibration:

1. Forms of equilibration that involve interactions between agent and environment and result from dis-equilibrium between coordination processes A, B, C, … and environmental aspects A′, B′, C′.
2. Those due to the reciprocal accommodation and assimilation *between* sensorimotor or cognitive schemes, so tensions due to the inability to assimilate or accommodate sequences or relationships of the form (A, B, C) ↔ (X, Y, Z) lead to new forms of adaptation (A_1_, B_1_, C_1_) ↔ (X_1_, Y_1_, Z_1_).
3. Forms of equilibration that result from tensions between a particular scheme and the system’s totality. This is, according to Piaget, a new form of equilibration, since it involves a hierarchical dimension of relationships among schemes or subsystems.

Piaget’s framework provides a progressive microgenetic conception of the changes that make cognitive development possible. This is important for the problem of perceptual learning. Conceiving of understanding as something that can only be either present or absent leads to the Platonic conundrum: I can only perceive what lies in front of me if I understand it with the categories and skills I already posses; yet new categorizations are required to perceive something new and there seems to be no source of categories other than those I had before. In contrast, a microgenetic approach works on a *graded* conception of understanding and so it allows us to specify the mechanisms and processes involved in the gradual emergence of new perceptual categories, habits, organized sensorimotor schemes and operations from pre-existing ones. Piaget’s development of the theory, particularly along its notation system, lacks a detailed dynamical systems formalization that can make justice to the microgenetic learning and equilibration processes he conceptualized.

## A dynamical approach to equilibration

In order to evaluate whether Piaget’s framework can serve as the missing learning theory for the SMC approach, our strategy is to describe Piaget’s ideas in modern dynamical systems terms and relate those with our previous dynamical definitions of SMCs (Buhrmann et al., 2013).

We do not propose here to offer a full dynamical systems account of Piaget’s theory of equilibration. But we will demonstrate how a dynamical systems interpretation of this theory, even if only partial, can act as a common language between Piaget and SMC theory. Both theories share the view that a perceptual situation is constituted by the coordinations that an agent is currently engaged in through its interaction with its surroundings (the deployment of skillful sensorimotor coping) and the possibilities for action that the situation makes possible for that particular agent and its skills or set of sensorimotor capacities. However, the point where SMC theory remains mostly silent is, as we have argued, exactly where Piaget has put his focus: the *transformation* of these coordinations and schemes. And, reciprocally, the sensorimotor approach has been formulated at a level of detail and attention to embodied and situated aspects of cognition that can complement some of Piaget’s proposals or suggest new interpretations.

We first recapitulate the dynamical systems interpretation of SMCs given in Buhrmann et al. (2013).

For the sensorimotor approach, perception relies on regularities in the sensorimotor flow, that is, on SMCs. The concept of SMCs refers to “lawful” co-variations of sensory stimulation and motor activity. For example, the projection of a horizontal line onto the retina changes from a straight line to a curved arc as one shifts the eye’s fixation point from the line itself to points above or below it. In contrast, if the focus is moved along the line no such transformation takes place. The geometry of the viewed object, the morphology of the retina, and the particular movement pattern employed, all determine regularities in sensory stimulation (O’Regan and Noë, 2001, p. 941). However, what counts as sensorimotor dependence varies if we focus on all possible scenarios given the details of the agent’s sensory and motor systems and its environment, or if we study the agent as the partial creator of such regularities, or if we consider different task-oriented scenarios.

To account for these possibilities, Buhrmann et al. (2013) propose four formal concepts of SMCs, and describe them in dynamical systems terms. These are: *sensorimotor environment*, *sensorimotor habitat*, *sensorimotor coordination*, and *sensorimotor strategy*. The sensorimotor environment is the set of possible sensory changes induced by arbitrary (open-loop) motor activity. It depends on the structure of the environment and the details of the agent’s embodiment, but not on the internal activity that regulates the agent’s behavior. It can be used to determine sensorimotor invariants such as relevant symmetries and asymmetries (e.g., the retina example). The sensorimotor habitat is again a general mapping, but taking into account the closed-loop situated agent, i.e., how the agent itself induces motor changes and how these changes affect sensory activity. The first two sensorimotor structures are general in the sense that they are supposed to map the full spectrum of possibilities for a given agent and situation. The next two sensorimotor structures are more specific. A sensorimotor coordination describes particular sensorimotor patterns that are reliably used in performing a task. These can be cycles or transients in sensorimotor space and depend on the environment, the body, the inner activity, and the task-related context. Finally, sensorimotor strategies are organizations of several sensorimotor coordinations, which are subject to some normative framework (for instance, considerations of efficiency or fluency).

These concepts are formalized as functional mappings involving variables such as the activity of sensors and motors, internal (neural) activity, relative positioning and configuration of the body, and so on.

In Piaget’s terminology, sensorimotor coordinations in the SMC sense correspond rather straightforwardly to the patterns of coordinations grouped under the labels A, B, C, … that form the organism’s side of the pairings that when organized in a cycle compose a sensorimotor sequence scheme or organization. The latter total scheme or organization corresponds to one kind of sensorimotor strategy according to the terminology of Buhrmann et al. (2013). The environmental responses A′, B′, C′, …, again in Piagetian terms, are also taken into account in the dynamical formulation of SMCs (ibid.) through an equation describing the environmental intrinsic and responsive dynamics. These and other aspects of terminology are summarized in Table 1.

**Table 1:** **Summary of Piagetian and dynamical systems concepts for a theory of equilibration**.

**Piagetian concept**	**DS definition**	**Notation**	**Example**
SM coordination scheme	Class of SM coordinations, defined e.g., by region in SM space, task constraints, etc.	A, B, C…	The class of movements and sensations that belong to the subject’s experience of pushing objects toward the ground; absorbing impacts with the hands etc.
Environmental response structure	Those environmental variables most directly affecting the sensory variables in A, B, C. i.e., the projection of the whole dynamic system, when engaged in SM coordinations A, B, C, onto relevant environmental variables.	A′, B′, C′…	Sound of the ball hitting the floor; height of the ball above ground; force exerted by the ball on the hand
SM coordination	Instance of SM coordination belonging to class A, B…, i.e., a trajectory in SM space that belongs to the respective SM coordination class.	*a*(*t*), *b*(*t*),…	A particular instance of pushing the ball towards the ground
Environmental response	Instance of environmental response of class A, B…	*a′*(*t*), *b′*(*t*), …	The sound of the impact for this particular bounce
SM coordination and environmental response tuple	Simultaneous occurrence of SM coordination *a*(*t*) ∈ A and corresponding environmental trajectory *a′*(*t*) ∈ A′ in the coupled system.	<*a, a′*>	
	The set of all tuples <*a, a′*>.	A × A′	
Sensorimotor organization or sequence scheme	Sensorimotor strategy. A sequence of SM coordination classes (and their corresponding environmental projections).	**O**: A × A′ → B × B′ → … → A × A′	Ball bouncing sequence of coordinations that includes pushing the object towards the ground, hearing the impact, waiting for its return, preparing muscles for contact, absorbing the impact and pushing it back.
Assimilation of A′ by A in **O**	(1) S*tability condition*: all *a′*(*t*) ∈ A′ are environmental responses corresponding to SM coordinations *a*(*t*) ∈ A. Or simply: all *a*(*t*) are true SM coordinations. (2) *Transition condition*: all *a*(*t*) ∈ A are special SM coordinations, namely reliable *transients* leading to the next scheme in **O** (e.g., B).		Continuous, stable ball bouncing despite small variations in motor pattern or wind speeds
Accommodation of Y′ into **O** by A	Parametric changes that re-establish a closed set of schemes **O** or **O**_1_ such that Y′ becomes the environmental projection of the whole system when engaged in A, which is a scheme belonging to organization **O**. This can involve modifying the previous A or creating a new scheme A_1_ and integrating it into **O**.		Learning to bounce a ball on a slope
**Lacuna**: perturbation of SM scheme due to a “gap” in understanding	Violation of the *transition condition*. Something is manifestly “unknown” about the world, since the presumed “right” handling of the situation (A × A′) does not lead to the next stage in the cycle (B × B′).		Bouncing a ball on a slope for the first time. Ball does not return to the same position.
**Obstacle**: perturbation of SM scheme due to contradictions and disturbances.	Violation of the *stability condition*. Something in the sensorimotor coordination has failed where in the past it used to work.		Attempting to bounce a new ball that is significantly heavier than the one that had been accommodated. Bouncing demands more strength.
**Equilibration**: ongoing adaptive process involving assimilation and accommodation that stabilizes the totality of SM schemes against perturbations by an ever changing environment (lacunae, obstacles) and internal tensions.	A potentially never ending series of parametric changes of the totality of SM organization, aimed at maximizing the stability of each organization against violations of the transition and stability conditions resulting from environmental perturbations or internal tensions.		The process of learning to bounce the ball under a variety of conditions (size and weight of the ball, slope and friction of the floor, etc.).

Although comparable with Piaget’s cyclic organizations, sensorimotor strategies also refer to more detailed aspects of sensorimotor order that remain implicit in the theory of equilibration. For example, sensorimotor strategies need not present a circular organization at the level of sensorimotor coordinations. This is because their normativity can be grounded elsewhere, either in the self-constitution of the organism in the enactive approach (Di Paolo, 2005; Thompson, 2007; Di Paolo et al., 2010) or in norms originating externally that the organism incorporates (efficiency in labor time, craftsmanship, performance, etc.). For these reasons, sensorimotor strategies can be more complex than cycles, but it is nevertheless possible to apply the notions of assimilation and accommodation by adopting a criterion of equilibration that follows the organismic or the externally imposed norm.

Barring these differences, that might later be exploited to inform the Piagetian approach, it seems so far that Buhrmann et al.’s (2013) dynamical approach to SMCs promises to establish a compatibility between equilibration and SMC theories. What remains to be seen is how we interpret the concepts of assimilation, accommodation, and maximal equilibration in these terms. First, we look again at equilibration, describing it as simply as possible in dynamical terms.

The agent and the environment are two coupled systems, i.e., some parameters in each of these systems are affected by the state of at least some variables in the other. Throughout the following analysis we will focus on two lower dimensional projections of the full coupled system: one projection looking at the state of the agent’s sensorimotor variables (S and M), and another projection looking at the sub-set of environmental variables that have a direct effect on the agent (E). The variables in the sensorimotor projections are affected by other variables belonging to the agent as well as by variables in the environmental projection. And similarly for the environment. In other words, these projections do *not* describe the whole agent-environment system. Those agent variables (say hormonal state, neural activity) that are not directly coupled to the environment, and similarly the environmental variables that are not directly coupled to the agent, are not expressed explicitly, though of course they may impact the sensorimotor process.

Let us consider an equilibrated sensorimotor organization, which we will denote as **O** = A × A′ → B × B′ → C × C′ → A × A′, where the notation A × A′ indicates a combined state in the cycle involving sensorimotor variables in A and the corresponding co-occurring environmental variables in A′. Dynamically speaking, each class of sensorimotor coordination (A, B, C) involves establishing structured patterns of motor and sensor co-variation in a task-related context. These can take the form of a reliable transient (one that will likely occur in the right conditions) or a metastable set of states *a*(*t*) that fulfils the condition of belonging to the same sensorimotor class, i.e., *a*(*t*) ∈ A. The class A is defined as those sensorimotor trajectories that assimilate those aspects of the environment that contribute to generating trajectories that belong to A′. We now clarify what this means.

**Figure 1:**
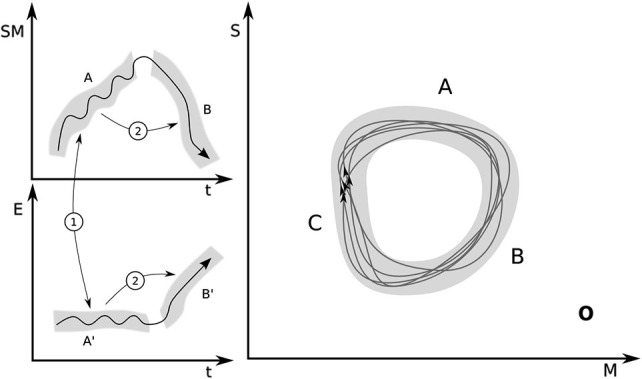
**Left**: Illustration of the two conditions describing assimilation. Condition 1: stability. A trajectory *a*(*t*) in the projection of sensorimotor space (SM) belongs to a set A (sets are represented by gray bands), which is mutually stabilized in coupling with a trajectory in the relevant projection (E) of environmental variables *a*′(*t*) that belongs to region A′. In other words, the SM trajectories in the upper panel and the environmental trajectories in the lower panel are both projections of the whole coupled system onto the respective subspaces during SM engagements of type A and B. Condition 2: transition. Trajectory *a*(*t*) in coupling with *a*′(*t*) lead respectively to *b*(*t*) ∈ B and *b*′(*t*) ∈ B′, the next stage in the sensorimotor organization **O**. **Right**: Projection of **O** onto sensory (S) and motor (M) coordinates.

We shall say that a sensorimotor coordination in class A assimilates an environmental feature or process that contributes to producing environmental time-varying states *a*′ ∈ A′ when the following two conditions apply (see Figure 1, left):

1. *Stability condition*: a sensorimotor pattern *a* = *a*(*t*), *a* ∈ A occurring in conjunction with an environmental pattern *a*′ = *a*′(*t*), *a*′ ∈ A′ are mutually stabilized, i.e., the full agent-environment coupling does not produce sensorimotor or environmental states outside the respective sets.
2. *Transition condition*: if any combination of trajectories *a* and *a*′ in the coupled system is produced such that *a* ∈ A and *a*′ ∈ A′, then this leads in time to the production of sensorimotor pattern *b* = *b*(*t*), *b* ∈ B in the agent and the production of states *b*′ = *b*′(*t*), *b*′ ∈ B′ in the environment, where B × B′ is the next stage in the cycle.

These conditions are then applicable to other links in the chain, so that as the agent approaches a state in B and the environment approaches some state in B′, these states tend to stabilize each other (condition 1) and lead the coupled system to the next stage (condition 2).

As a shorthand we will sometimes describe the class of sensorimotor coordinations A as *assimilating* the class of environmental features A′.

Notice that because the dynamical systems description is formulated without assuming a clear delimitation into well-defined agent or environmental stages (A × A′ → B × B′ →), it is more general than the typically staged Piagetian style (A × A′ → B; B × B′ →) (Piaget, 1975, p. 10). In other words, since the dynamical interpretation assumes a necessary coupling between agent and environment, neither behavioral (as suggested by Piaget’s notation) nor environmental consequences alone are taken to be solely responsible for transitioning to the next SM coordination within a given SM organization. The Piagetian case is a particular version of the dynamical description where the coupled system produces a clear behavioral outcome *b* ∈ B which is later co-responsible (in combination with the environment’s intrinsic dynamics) for the production of a state b′ belonging to the assimilated environmental set B′. This staged mode does not need to occur in all circumstances nor is it essential for interpreting assimilation dynamically.

Graphically the sensorimotor organization **O** can be represented as a set of closed loops when the coupled system is projected onto the space of sensorimotor coordinates (Figure 1, right). Each loop is not necessarily identical to the others because assimilation implies that the agent’s sensorimotor activity will keep on cycling through the equivalent sensorimotor states *a* ∈ A, *b* ∈ B, and *c* ∈ C in equilibrated coupling via conditions 1 and 2, with environmental states *a*′ ∈ A′, *b*′ ∈ B′ and *c*′ ∈ C′ respectively (Figure 1, left). We take the bundle of all these possible trajectories as the graphical description of the cycle **O**. The gray areas then represent the different sets A, B, C (and A′, B′ in Figure 1 left). In the dynamical formulation, the distinction between stages must somehow be pre-given and related to meaningfully distinct (at least from the agent’s or the observer’s perspective) sensorimotor engagements (suckling, swallowing, breathing, etc.). In other words, the difference between sensorimotor engagements A and B is defined externally to what is shown in this figure. The shape and extent of the gray areas, however, is defined by meeting the conditions 1 and 2 in a way that a closed cycle is formed (Notice that the gray areas are drawn as smooth and continuous for illustration purposes; in general the sets A, B, … and A′, B′, … need not have obvious topological properties in sensorimotor coordinates). In dynamical terms, in this representation, the organization **O** corresponds to a metastable region in sensorimotor space. We assume that equilibration has been maximized if cycles occur within the gray band.

Let us now consider accommodation. A perturbation to the sensorimotor organization **O** implies that a situation has occurred that locally, at some point in the cycle, does not fulfill either condition 1 or 2. In these cases the environmental dynamics are not assimilated by the agent.

This can happen, for instance, when the stability condition (1) fails: during the A × A′ situation, and for reasons that can originate in internal or environmental processes, the sensorimotor trajectory *a* ∈ A and the environmental trajectory *a*′ ∈ A′, or both, become unstable (i.e., they fall outside their respective assimilated sets). This leads either the agent or the environment, or both, to trajectories outside the assimilated sets: *d* ∉ A and/or *d*′ ∉ A′. The agent experiences this as an *obstacle*; something in the relation between environmental variables and the enacted sensorimotor coordination has failed where in the past it used to work.

Alternatively, the perturbation may occur when the transition to the next stage in the cycle fails (condition 2). Even if *a* ∈ A and *a*′ ∈ A′ are both within their respective sets A and A′, the conditions of the coupling change such that instead of leading to *b* ∈ B they lead to *e* ∉ B and/or instead of leading to *b*′ ∈ B′ they lead to *e*′ ∉ B′. This is the case of a *lacuna*, i.e., something is manifestly unknown about the world since the presumed “right” handling of the situation (A × A′) does not lead “as expected” to the next stage in the cycle.

Notice that the dynamical interpretation is based on the properties of the agent-environment coupling, i.e., on the *relation* between the agent and the environment. Then a particular failure (in conditions 1 or 2) can in principle originate from either internal or environmental proximal causes. The origin of a perturbation is invisible to the agent, only its effect is manifested as a disruption of the sensorimotor scheme: the loss of “control” over a previously stable sensorimotor coupling or the failure of an effectively achieved coupling to lead to its usual result. The terms obstacle and lacuna are used here for their relational effects on action and perception, not for their (not directly perceivable) proximal causes.

If we assume that the gray areas in Figure 1 represent trajectories belonging to the condition of having achieved maximal equilibrium and that these trajectories define the subsets A, B, and C (and a similar condition of maximal equilibration defines the sets A′, B′ and C′ on the environmental side), then any perturbation as defined above (either an obstacle or lacuna) will make sensorimotor and environmental trajectories escape from their sets of maximal equilibration (the gray zone). And, all other things remaining equal, in principle, it cannot be expected to return to the gray zone, except fortuitously, for instance, through an independent environmental change. Anything that at the personal level could be described as an attempt to deal with an obstacle or lacuna, i.e., an attempt to bring the unexpected situation back into the sensorimotor organization **O**, will imply at the dynamical, subpersonal level that things do *not* remain equal—i.e., that some form of plastic change must occur.

Both the agent and the environment, as dynamical systems, are described by a set of variables and a set of constraints and parameters. We call this latter set R for the agent and R′ for the environment. A coupling, as we have said, implies that at least some of the parameters in one system depend on the state of the variables in the other. But other processes apart from the coupling may also drive parametrical changes and we refer to these as processes of explicit plasticity.

For accommodation to occur in the conditions we have described, some form of explicit plasticity is needed. Contrary to what is traditionally assumed, it is possible for a system to exhibit adaptation, learning and other history-dependent behaviors without explicit plasticity (e.g., Izquierdo et al., 2008). Those forms of adaptive behavior rely on the rich dynamical possibilities of systems with sufficiently high dimensionality. Such systems learn by selecting different regions of their dynamical landscape in a history-dependent manner, a form of implicit “plasticity”. Thus, agents controlled by dynamical neural networks can perform some forms of learning and history-dependent categorization without any changes to the structure of these networks (e.g., to the connection weights). In our description, such systems would by themselves, without the need for parametrical change, already assimilate what may look like a perturbation at the local, immediate timescale. Externally, and with respect to the timescale of behavior the agent is seen as “perturbed” and then adapting to this “perturbation”. But on a sufficiently long timescale, in the absence of explicit plastic changes, a reliable (not fortuitous) return back into the sensorimotor scheme **O** implies that the original “perturbation” was not such, and had been assimilated all along, only that this did not seem to be the case at the timescale of observation.

In principle, plasticity may occur on the agent’s side or in the environment (either in R or R′). Let us denote as <*z*,*z*′> the simultaneous occurrence of sensorimotor trajectory *z* and environmental trajectory *z*′ *in the coupled system*. The set of all tuples <*z*,*z*′> corresponds to Piaget’s notation Z × Z′. Thus, by the notation <*z*,*z*′> ∈ Z × Z′ we simply mean that in addition to occurring simultaneously during coupling, *z* ∈ Z and *z*′ ∈ Z′. Consider, for instance, the case of a lacuna, a failure in the transition A × A′ → B × B′. This means that, after producing <*a*,*a*′> ∈ A × A′, a new combined state <*e*,*e*′> is produced where at least one of the following conditions is true: *e* ∉ B or *e*′ ∉ B′. This breaks down the cycle. Let’s suppose that, on attempting the same transition again, a plastic change has the effect of producing a different sensorimotor transformation <*a*,*a*′> → <*b*_1_,*b*_1_′> such that the new environmental trajectory *b*_1_′ is now produced instead of *e*′ and a new sensorimotor coordination *b*_1_ is produced instead of *e*. We assume that like *e*, *b*_1_ ∉ B or that like *e*′, *b*_1_′ ∉ B′, i.e., either the new sensorimotor trajectory or the new environmental trajectory, or both, are still outside the previously assimilated set. However, let’s assume that unlike the combination <*e*,*e*′> now the combination <*b*_1_,*b*_1_′> does lead back to <*c*,*c*′> ∈ C × C′. Then the factors that lead to the new trajectory *b*_1_′ have been accommodated. If the accommodation does not disturb the already assimilated set B′ (which may or may not be the case), then the set B′_1_ = B′ ∪ *b*_1_′ defines the newly assimilated environmental conditions and *B*_1_ = *B* ∪ *b*_1_ the accommodating class of sensorimotor coordination. The new organization is now **O**_1_ = A × A′ → B_1_ × B_1_′ → C × C′ → A × A′. In longer sensorimotor schemes a return to the cycle may occur at a later point in which case the sets describing the intermediate links need to be redefined accordingly. The case of an obstacle can be treated similarly (plasticity would be involved in transforming the new situation <*e*,*e*′> into <*b*_1_, *b*′_1_> such that *b*_1_ and *b*_1_′ reliably stabilize each other within the accommodated new set B_1_ × B_1_′).

In Piagetian descriptions, accommodation seems always to imply plastic changes in the agent and not in the environment. This is fitting since Piaget is interested in human development starting from its biological roots. However, it is interesting to note that we often equilibrate our sensorimotor operations by modifying the environment (or indeed that other agents contribute to our equilibration). Like most species we are active constructors of our environments, either purposefully or not. If it is too cold to perform our activities outdoors, we accommodate this obstacle by wearing warmer clothes, not by growing fur. And there may be a range of less obvious cases where the agent’s activity is not directly aimed at transforming the environment but such transformations occur nonetheless—e.g., the formation of trails on grass, the emergent spatial ordering of work spaces (Agre, 1997; Kirsh, 1995, 1996), and so on. In the rest of this section we stay on the agent’s side to keep things simple and because it fits the traditional Piagetian interpretation. However, the analysis permits equally well the consideration of cases in which accommodation occurs by the agent inducing environmental plasticity such that the sensorimotor patterns are modified without requiring any physical alteration to the organization of the agent itself.

How should changes in the set of parameters *R* occur? What triggers them? These are important questions that will largely depend on the case in question. Ashby’s (1960) general formulation postulates that these changes could happen at random as soon as they are triggered by the mismatch between the current situation and the acceptable (equilibrated) set of possible agent-environment states. In such a scheme, random changes in the parameters *R* governing the agent’s sensorimotor coupling with the world would lead to exploration of the space of possible SM coordinations, and this process would terminate only when re-equilibration is achieved. It is clear that natural adaptive behavior uses more sophisticated strategies about which we can say little in general terms here. But we can affirm one implication that arises from our formal description: accommodation always involves an element of randomness. If this was not the case perturbations could not occur since we have assumed maximal equilibration *ex hypothesi*. Moreover, if a sure, deterministic accommodating strategy exists and the agent can deploy such a strategy, this means that the “perturbation” had been assimilated all along as the closure of the cycle was guaranteed (although by external standards it may look as if the agent is struggling to accommodate a new environmental feature). Therefore, open-ended accommodation implies some degree of random search in how internal parameters are affected and/or randomness in how the environment responds to these parametrical changes.

If and when accommodation has only additive effects, i.e., they add to the set of assimilated states without subtracting previously assimilated conditions from the previous sets, then maximal equilibration is conserved. If not, maximal equilibration may be re-attained through a sequence of accommodation steps (learning the new but also re-learning the old). One might assume that the meta-stable situation that defines the sensorimotor organization would imply a tendency towards maximizing equilibration. This is not necessarily the case and will depend on the strategy used to regulate plasticity.

In practice, in many cases we witness a tendency towards maximal equilibration as learning progresses. This tendency can be measured by dynamical signatures, for instance by studying long terms correlations (e.g., van Orden et al., 2003; Dotov et al., 2010). Such measures are indicative of the degree of fluency in sensorimotor engagements and other important aspects, for instance whether the action is more driven by the agent or by the environment.

Earlier, we have linked the Piagetian sensorimotor organization or scheme with the dynamical concept of sensorimotor strategy (Buhrmann et al., 2013). The latter, as we have said, allows for complex spatiotemporal relations between partial sensorimotor coordinations, which in Piagetian terms would be subsumed as a sequence of states in a totality. Without radically altering the present analysis, we can account for Piaget’s second type of equilibration (between sensorimotor coordinations), by simply noting that plasticity in the agent may occur not only in parameters that regulate the sensorimotor coordinations themselves, but also their inter-relation as defined by a sensorimotor strategy (e.g., relative timing, duration and intensity). In fact, it seems unlikely that in complex adaptive systems, a parametrical change would affect only one sensorimotor coordination without affecting others. The condition of equilibration in such cases would not necessarily be a return to some later segment of the original cycle, but a mutual accommodation of the various elements of the sensorimotor strategy with respect to each other; a transformation of the scheme as a whole.

**Figure 2:**
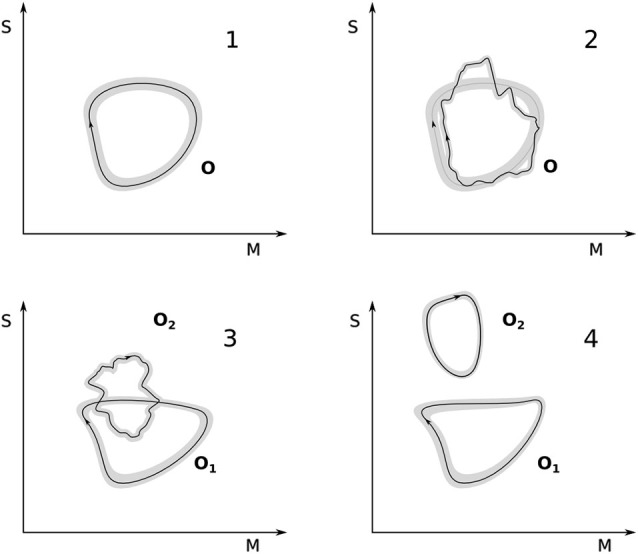
**Accommodation and equilibration**. Top left: a maximally equilibrated sensorimotor organization **O**; a trajectory in sensorimotor space is shown within the gray band defined by **O**. Top right: a perturbation brings the sensorimotor trajectory outside the gray band, however, due to plastic changes, the trajectory is accommodated and a cycle restored. Bottom left: through repeated perturbations and re-equilibrations, the organization **O** has been turned into **O**_1_ and another organization **O**_2_ has been equilibrated, they correspond now to two different kinds of sensorimotor engagements. Bottom right: **O**_2_ attains maximal equilibration (represented graphically as a smoother trajectory that does not leave the gray band) and **O**_1_ has been re-shaped due to a mutual accommodation between the two clearly distinct sensorimotor schemes.

Figure 2 illustrates the foregoing analysis. Panel 1 shows a maximally equilibrated organization **O** and one given trajectory within the gray zone that defines it. A perturbation occurs (panel 2) such that the coupled system moves away from the maximally equilibrated area. A series of plastic changes are induced with the result that the trajectory comes back in some areas to the original cycle. As further accommodation events occur the new scheme **O**_1_ may become maximally equilibrated again (panel 3). Here we also show an additional possibility. It may happen that new metastable regions can be discovered by the plastic exploration of sensorimotor couplings while the system is in the process of accommodating the original perturbation. This will result in the creation of a new sensorimotor organization **O**_2_ without the disappearance of the modified original one **O**_1_. This organization may not be initially maximally equilibrated but, in the right circumstances may increasingly approach this condition. This difference is graphically illustrated as the difference between the jagged loop in panel 3 and the smooth loop in panel 4 (although this is merely a convention; in some cases equilibration could indeed look jagged in the plot and still be maximal).

In terms of our previous example, **O** could correspond to the sensorimotor breastfeeding cycle (Figure 2, panel 1). A perturbation occurs (panel 2), for instance, the first attempt at feeding from a milk bottle. The breastfeeding cycle serves here as the departure point for accommodating the new situation. Through plastic changes and “re-use” or adjustment of sensorimotor elements in the breastfeeding cycle (e.g., the baby changing the relative timing, duration and intensity of suckling, swallowing and breathing), the milk bottle is accommodated for the first time. Through further disequilibrium and subsequent accommodation, the milk bottle cycle begins to be maximally accommodated. A separate gray area begins to be defined (panel 3), which corresponds to new milk-bottle feeding sensorimotor scheme **O**_2_. The breastfeeding scheme remains, though possibly modified in shape (**O**_1_). There may be some “interaction” between the two organizations (higher order equilibrations as described by Piaget) through mutual accommodation. **O**_1_ is likely to be shaped differently, as **O**_2_ becomes maximally equilibrated (panel 4) but the details of this higher-level plastic process are not described here.

We now come back to the example mentioned earlier of the visual inversions studied by Kohler (1951 [1964]), and try to interpret his behavioral and phenomenological reports in terms of processes of equilibration (though for reasons of space not in the amount of detail this exercise would merit). Kohler’s main finding was that re-adaptation of the visual field always occurs only partially and step-wise. The first successful adaptations, required it seems for any subsequent correction of the visual experience, are always those involving co-occurring non-visual (e.g., tactile) sensations and/or overt bodily interactions. Thus walking, avoiding obstacles, reaching etc. become increasingly better and eventually almost perfect, while the visual experience itself is still judged to be non-veridical. After such skills are recovered, this is usually followed by recognition of objects that can be “brought into a new behavioral relationship with one’s body” (ibid. p. 158), such as one’s face seen in the mirror, or one’s shadow projected on the floor. Adaptation of visual experience is equally fragmented: objects might appear in the correct place, yet remain mirrored in orientation (the position and direction of movement of a car is perceived correctly, while the letters on its license plate remain reversed). This fragmentation of the skill of seeing led Kohler to interpret his experiments as a means to probe the transformation of “the structure and mutual interweaving of [perceptual] habits” (ibid. p. 139), and the process of adaptation as one of rehabituation.

Identifying “perceptual habits” (ibid. p. 140) with sensorimotor strategies and rehabituation with equilibration allows us to find further similarities. Firstly, new attempts at equilibration always depart from what is already known, i.e., from existing SM coordinations. During rehabituation this results in a failure to grasp objects or move in the intended direction, since there is now a mismatch (lacuna or obstable in Piagetian terms) between vision, proprioception and intended movement. Interestingly, conscious strategies for controlling (compensating) fast grasping movements are not successful or stable in the long run. Kohler reports that in the end only repeated and essentially random reach attempts eventually lead to the gradual adjustment of movements and the co-occuring visual experience, supporting our requirement for randomness in the exploration of new SM coordinations. Secondly, adaptations always seem to be task-specific, i.e., progressive trials are guided towards achieving closure of some kind (e.g., reaching the object). Equilibration also occurs gradually. The first cases of success are often unstable until equilibration is maximized and new metastable SM schemes are fully formed. There also exists a “particular splitting in the difficulty or ease of correct mastery” (ibid. p. 144) of different visually-guided actions: equilibration in one task does not transfer to others. The kind of left-right perceptual habits involved in walking or cycling are different from those used in reading letters, for example. Each perceptual skill is mastered by undergoing an equilibration process of its own. And only when all tensions between individual SM schemes are resolved, i.e., after appropriate re-equilibration (or equilibration of the second and third category), is the world as a whole perceived as coherent, i.e., correct vision re-established (though this may never be fully achieved in practice).

Another useful example is learning to bounce a ball under different conditions. This example and a summary of the different dynamical concepts introduced appear in Table 1.

## Principles for sensorimotor equilibration

Even though the language of equilibration might seem rather specific, the dynamical interpretation provided earlier allows us to extract a number of general principles and requirements that we believe apply to any approach to learning new sensorimotor categories in an open-ended manner.

To begin with, SM coordinations form *equivalence classes*. Our definition of the stability and transition conditions for assimilation implies the possibility for different SM coordinations to contribute to the closure of a SM organization. The equivalence of SM coordinations need not be identifiable topologically (in SM space). Instead, it is established solely by their playing equal roles in the completion of a sensorimotor task.

Learning agents need to be able to quickly respond to varying environmental situations. This requires that they never reach stable equilibrium. SM coordinations, therefore, must only be temporarily “stable”, or *meta-stable*. This is accounted for in our requirement that each SM coordination leads naturally to another, i.e., in the transition condition (and similarly it must also be possible to move between higher-level SM organizations in an itinerant manner). Reliable SM organizations, given their dependence on equivalent classes of SM coordinations, and not on specific SM trajectories, are therefore “stable” not in the mathematical sense, but internally coherent: i.e., they lead to the continued renewal of the SM organization itself (in the case analyzed here, the SM organization loop or scheme).

Also, in order to respond appropriately to different environmental contexts, *mechanisms of selection* are needed to choose which SM scheme to enact in a given situation. Random selection might be necessary in situations never encountered before, and might underlie the exploration of new SM coordinations during equilibration. But more directed choices could develop as well. This need not be based on abstract deliberation or decision-making. Dynamical mechanisms that allow environmental conditions to “call for” a certain SM engagement are also imaginable (Buhrmann and Di Paolo, 2014). Also, in the same way that certain SM coordinations naturally follow each other within a circular organization, there might be propensities for some organizations to be followed by certain others. For instance, a sucking reflex might usually be preceded by an organization that guides the baby’s attention in the necessary direction, like the rooting reflex during the first 4 months, which in turn might be preceded by an organization that seeks proximity to the mother and so on. Another possibility might involve the similarity of SM schemes (and/or environmental conditions), such that similar but different schemes can be tried in similar contexts. This would also work through hierarchical organization of SM schemes without requiring a quantitative similarity measure. For example, if through accommodation a new bottle-sucking scheme is established that in turn derives from a previous breast-feeding scheme, then both schemes together could form a higher-level class of sucking schemes.

Accommodation involves *normative evaluation* of SM schemes. Firstly, this is required for assessing when an assimilation attempt has failed and whether adaptive processes need to be triggered. Secondly, in the process of accommodation, new engagements need to be evaluated as better or worse than the failed one, in order to determine which adaptations to fixate in the system. There may be multiple sources of normative evaluation, including external ones, consider the situation of an apprentice being guided to improve her movements. But, as a principle, it is necessary to have a least some internal sources of normativity (e.g., survival, closure of SM scheme) in order for the agent to evaluate situations in which externally imposed norms (e.g., a designer’s utility function or value system) break down or cease to apply. An *exclusive* reliance on external norms and value systems in models of learning by definition imposes limitations on the universe of learnable behaviors (i.e., they are restricted to those possibilities considered by the externally imposed value system) and therefore are an impediment to open-ended learning.

Accommodation also requires *adaptive mechanisms* for modifying existing SM coordinations. An important Piagetian principle is that accommodation always starts from some pre-existing sensorimotor scheme, which undergoes adaptation if it cannot assimilate a given environmental feature. In order to achieve this goal, random “mutations” (i.e., generation of new potential SM schemes) are in principle required for true open-ended learning (in addition to more guided forms of learning). Otherwise, a system cannot, even potentially, transcend its current “laws” of operation. The randomness in question need not involve complete, but only partial, statistical independence from the previous state (see also Campbell, 1974). “Motor babbling” is one example of randomness creating new interactions with the environment. But while randomness is required for true open-ended learning, it will typically not be the most efficient route to learning in those cases which are recognizably similar to what has been encountered before. In such cases, a perceiver can also learn from *the way* she fails. Directed learning could rely, for instance, on gradients in the normative evaluation of SMCs, or on the discrepancy between a scheme’s actual and expected outcomes.

Finally, in a complex system involving many different SM organizations, e.g., in the case of hierarchically organized SM structures, the accommodation of one scheme might interfere with others established previously (analogous to the stability-plasticity dilemma in traditional learning theory). Not only must accommodation produce valid SM schemes, but these are also subject to a more global coherence constraint. In other words, equilibration does not only involve adaptation of individual SM schemes, but also the *re-equilibration* of the SM repertoire as a whole.

In sum, the building blocks of open-ended learning, according to the approach presented here, are meaningful SM coordinations (rather than, say, individual SM states). We learn to perceive and interact with something never before encountered through equilibration of an organization of such SM coordinations. Through a process of incremental, adaptive differentiation of existing SM coordinations—bootstrapped by simple sensorimotor loops either already present at the earliest stages of development or discovered through self-organizing processes (e.g., Marques et al., 2013)—previously established SM know-how is adapted to a new context, or new patterns of interaction are generated and integrated with an already existing set of SM schemes.

## Discussion

At this point we have established two important results. Firstly, Piaget’s theory of equilibration can be formulated in dynamical systems terms, and in those terms it is compatible with the operational notions of dynamic SMCs established previously by Buhrmann et al. (2013). Secondly, this dynamical version of Piaget’s ideas furnishes SMC theory with its missing theory of learning, and from there, with the chance to keep developing its conceptual primitive of “mastery”. In this section, we discuss two general implications of these results, i.e., implications of the principles gleaned from the dynamical formulation and implications for the personal-level experience of perceptual learning.

### Implications for open-ended learning

Several of the principles for open-ended learning discussed above are already present in other approaches. Ashby, for example, in his concept of ultrastability (Ashby, 1960), formulated perhaps the first mechanistic account of open-ended learning, namely as the random exploration of a large space of sensorimotor loops with the aim of achieving homeostatic equilibrium (for use of this idea in more recent work also see Di Paolo, 2000, 2003, 2010; Harvey et al., 2005; Iizuka and Di Paolo, 2007, 2008; Di Paolo and Iizuka, 2008; Manicka and Di Paolo, 2009; Izquierdo et al., 2013). Parallels with reinforcement learning (Sutton and Barto, 2009) and related sensorimotor approaches (e.g., Duff et al., 2011; Maye and Engel, 2011, 2013) can be drawn as well. For instance, the exploration-exploitation trade-off characteristic of such approaches is related to the assimilation-accommodation dichotomy in equilibration; and the global equilibrium towards which these systems tend is one of maximum expected reward, in analogy with the state of maximum equilibration.

However, equilibration differs from these approaches in crucial ways. Ashby’s (1960) ultrastability, for example, fails to account for more directed and efficient types of adaptation (other than randomness), and, at least in its original formulation, is at odds with the incremental nature of equilibration, according to which learning always starts from where you are now and tends to conserve previously learned behavior for small accommodations. Piaget’s account also distinguishes itself from other approaches in that learning is not seen as the discovery of existing structure in a pre-given space of perception-action states. It is rather the open-ended, combinatorial-like construction of new SM coordinations in an ever growing space of possible SM coordinations. Equilibration is thus more akin to the evolutionary radiation of species that leads to the ever-branching phylogenetic tree, or to selectionist adaptation in the immune system, which enables production of new antibodies for virtually every possible antigen. As Kauffman has noted (Kauffman, 2002; Longo et al., 2012), the space of solutions created by such mechanisms is “unprestatable”, in the sense that one cannot ahead of time determine the set of all possible SM coordinations a person might produce in the course of her life.

This open-endedness of Piagetian equilibration is in part due to the fact that most SM engagements will only become available in a history-dependent manner, when other SM engagements have been discovered that they depend on (like pre-adaptations in evolution). Very likely such a system will be non-ergodic (see also Kauffman, 2002). It will only ever visit such a small part of its “state space”, i.e., produce only the smallest number of SM coordinations out of all possible ones, that it would be on a unique trajectory (though commonalities in embodiment, social constraints etc. might limit this space somewhat).

Additionally, equilibration is open-ended because the world itself provides an open-ended repertoire of possible “behavioral niches”. There is no predictable end to the variety of physical couplings offered by the world. This is to be highlighted because, as we have argued, the world is a constitutive part of SM coordinations. It is not the agent’s learning architecture that is open-ended *per se*, but only in virtue of its capacity of coupling to an open-ended world. This is a point which can be seen more clearly in a dynamical analysis of SMCs, rather than in an account based on manipulation of internal representational states. In the dynamical perspective, the world plays a role in learning which is different from that of providing inputs to internal processing. Nothing in the formalism prevents aspects of the dynamics of the world forming constitutive parts of the learnt sensorimotor schemes, and indeed this is exactly what we see happening in implemented models. Aguilera et al. (2013) provide a strong example of the difference between world-coupling and world-as-input. Similar effects have been registered in models of social coupling (Di Paolo et al., 2008) analogous to situations like the double TV-monitor experiment by Murray and Trevarthen (1985) (see De Jaegher et al., 2010 for the social cognition implications of these models analogous to the point we are making here).

It should be noted, that at this stage the dynamical systems approach to sensorimotor equilibration is not a fully developed theory. It outlines the essential elements that such a theory will eventually have to contain, but several details, for example regarding its possible implementation, have yet to be filled in. Progress in this area will need to involve further work on the nature of open-ended learning, for instance further examination of the processes assumed to be open-ended in nature (such as biological evolution and the adaptive immune system) and their relation to processes that could be operating in the brain (e.g., Edelman, 1987; Calvin, 1997; Fernando et al., 2012).

Future work should also be aimed at identifying empirical evidence supporting the dynamical formulation of SM equilibration. In this regard, the history-dependent nature of this process suggests that it is necessary to study individual subjects’ learning trajectories as a function of their pre-existing behavioral repertoire. A good example is the study by Kostrubiec et al. (2012), in which dynamical systems analysis is used to describe different subjects’ strategies in learning a new sensorimotor skill. The authors find that the routes of learning, i.e., the dynamical adaptations involved, depend on the relevant SM coordinations each individual learner brings to the learning task. The observed adaptations are either small incremental modifications of an existing SM coordination, if it is similar enough to the coordination that is to be learned, or otherwise abrupt bifurcations that qualitatively change the underlying SM repertoire and create novel forms of SM coordination. In further support of the equilibration approach, the authors also show that the stability of the desired coordination, rather than detected errors in performance, can serve to guide sensorimotor learning. In general, new methods of investigation might be needed to study the development of non-ergodic systems whose qualitative properties change over time (see e.g., Molenaar and Campbell, 2009; Medaglia et al., 2011).

### Perceptual learning at the personal level

The dictionary notion of “mastery” is ambiguous. On the one hand, it can refer to “comprehensive knowledge or skill”; on the other, it can refer to the process of mastering such a skill. We have given an operational account of how sensorimotor schemes emerge and get transformed through the process of equilibration. How does this relate to both notions of mastery? The first thing to notice is that mastery as used in SMC theory is a personal level notion, i.e., achieving skills in accord with personal level norms. Therefore, to fully address this issue we need to investigate the links between the dynamical approach to equilibration and the personal level. This task is part of a larger project, which we cannot fully address here, but nevertheless we wish to suggest some links.

A further issue at the personal level is raised by our initial way of posing the problem of perceptual learning in relation to understanding. Understanding is something done by whole agents. For example, we have emphasized above that, in mastering the modified SMCs of inverting goggles, the subject needs to be involved in working out and understanding their new situation. Once again, we see that a full answer to this issue will involve working out an extended account, which explains how the norms and goals of a whole agent arise from multiple, interdependent sensorimotor structures (Beaton, 2013, 2014).

Here, we wish to make two points as regards further work on these two issues. Firstly, we believe that a full, personal story is compatible with the framework presented here. Secondly, we think that looking at what we cannot yet say about the whole-agent story helps to highlight where future work can be carried out in the operational story. In particular, we note that a real subject does not consist of one, or a few, sensorimotor loops as studied above, but instead has a vast number of interacting sensorimotor abilities, all at different levels of complexity, and which must all eventually be grounded in biological viability.

These issues are touched on in Piaget’s work, where he talks about accommodation and assimilation between different schemes of understanding, and also about the accommodation and assimilation between a given scheme and the whole framework of understanding of the agent. However (see e.g., Boom, 2009, p.138), Piaget never explored these second and third types of equilibration in anything like the detail in which he explored equilibration of his first type, namely between a given sensorimotor scheme and the world. Similarly, in the present work, we ourselves have so far considered only sensorimotor equilibration of a given sensorimotor scheme (a loop which perhaps splits into two), and we have not considered the different levels of stability that arise when multiple sensorimotor schemes are in interaction. To deal with learning at the level of a whole agent, these much more complex interactions must eventually be addressed.

Is the only question here one of complexity, or are other issues raised? We note that according to the sensorimotor approach, perception is always informed not just by the enactment of a sensorimotor skill (i.e., a closed sensorimotor scheme) but also by the set of potential skills which the subject already possesses, which may be relevant to the current situation. Consider, for example, two agents each of which has a feeding sensorimotor scheme which, when analyzed operationally, have the same sensorimotor stages and structures (same SM coordinations A, B, C, leading to the same environmental responses A′, B′, C′, same transitions, and so on). One of these agents has only this behavior, the other has in addition a repertoire of many other skills which can be engaged in different contexts. The sensorimotor approach suggests that the structure of perception is different (potentially much richer) in the latter agent than the former even when they are both engaged in the same behavior. This suggests that further examining the relation between the operational and the personal levels is not simply a matter of complexity but that we can expect qualitative differences when considering the agent as a whole.

Returning to the issue of mastery, we suggest that the full-blown, personal level term will be closely related to Piaget’s concept of equilibration. Equilibration refers to the full process of closing a sensorimotor scheme, and then progressively “stabilizing” that closed loop against perturbations. Thus, equilibration itself shows a close analogy with the two meanings of mastery: having, and improving, a sensorimotor skill. However, we suggest that, if mastery is read as a personal level notion, then giving a full account of mastery will depend on two things: further operational work on the interaction between actual (enacted) and potential sensorimotor structures, and further operational work on the grounding of sensorimotor norms in the biological normativity of the agent as a whole (c.f. Noë, 2004, p. 230).

## Conclusion

We have proposed a conceptual framework for understanding open-ended perceptual learning in the context of a missing theory of learning for the sensorimotor approach to perception. The proposal is inspired by Piaget’s theory of equilibration, but has been given a novel dynamical treatment and formalization. The result is fully compatible with sensorimotor theory (including the previous dynamical analysis given by Buhrmann et al., 2013).

We can now propose precise answers to our opening questions at the operational level:

- By what mechanisms is mastery of SMCs acquired?
  Through the ongoing process of maximizing equilibration in SM schemes or more generally SM strategies.
- What counts as having acquired sufficient know-how of SMCs?
  What counts as mastery?
  To achieve a sufficiently equilibrated SM scheme (with most systematic perturbations accommodated).
- How is it possible to learn to perceive anything new if perception itself always relies on existing knowledge of SMCs, as the theory claims?
  Perceptual learning starts from existing SM schemes, which undergo a process of equilibration in novel conditions. This may result in a new SM scheme. Once equilibration is achieved it may proceed by further accommodation until full mastery is achieved.
- How do various sensorimotor organizations relate to each other in the same agent?
  Through higher-level processes of equilibration between SM schemes (as illustrated in Figure 2).
- What kind of cognitive organizational principles can help sustain SMCs in a flexible, open-ended manner?
  At least these principles: (1) the organization of SM coordinations in equivalence classes; (2) the meta-stability of SM coordination; (3) the existence of selection mechanisms to choosing SM schemes; (4) an intrinsic normative evaluation of the equilibration process; (5) the existence of sufficiently rich adaptive mechanisms for altering SM coordinations; and (6) a higher-order re-equilibration mechanism to organize the relation between various SM schemes.

This does not mean that there are not still several open issues for further research. For instance, one unresolved question involves figuring out which plasticity mechanisms underlie the different stages in equilibration. It should also be noted that we have examined these questions taking a bottom-up approach in which an autonomous agent is confronted with obstacles and lacunae and must establish some re-equilibration following intrinsic norms. This by no means implies that all learning is like this, especially human learning. In such cases we cannot ignore the effects of parental scaffolding, external norms, and the linguistic guidance of others, eventually turning into linguistic self-guidance. We expect, however, that the bottom-up approach can serve as a departure point—it will surely not exhaust the issue—for other dynamical theories of skill acquisition and expertise that make explicit the passage from higher-level, social, linguistic and reflexive rules and norms to corporeal intentionality and habits (e.g., Dreyfus, 2002; Ravaisson, 1838/2008; Merleau-Ponty, 1945/2012).

The proposed framework leads to specific principles required for open-ended learning. Again, we do not claim this list to be exhaustive. In particular, we have argued for the importance of intrinsic norms, and for the ability of a system to, in some sense, “transcend its own rules”. The Piagetian approach provides an entry point into both of these aspects of open-ended learning. On the one hand, it makes intrinsic norms explicit, in terms of the closure of the sensorimotor scheme. On the other hand, equilibration proceeds by re-shaping pre-existing structures in coupling with dynamical regularities from the world (some of them unknown by the agent). Thereby, a Piagetian agent is never strictly bound to what is already known, even if this is always its departure point.

Perceptual learning presents no paradox as soon as the required mastery is seen not as the accumulation of internal representations, whose relevance and applicability would escape the agent in an unknown context. Instead, mastery is a regulated openness to be coupled to the world and to be guided by it starting from what has worked in the past. Mastery involves as much the agent as the world as sources both of meta-stability and of novelty.

## Conflict of interest statement

The authors declare that the research was conducted in the absence of any commercial or financial relationships that could be construed as a potential conflict of interest.
